# Development and feasibility of a modified Fugl-Meyer lower extremity assessment for telerehabilitation: a pilot study

**DOI:** 10.1186/s40814-021-00862-8

**Published:** 2021-06-07

**Authors:** Sue Peters, Marcela Botero, Allison Evers, Brianna Fong, Botond Jakab, Emily Petter, Janice J. Eng

**Affiliations:** 1 Elborn College, Room 1000, Western University, 1151 Richmond Street London, Ontario, N6A 3K7 Canada; 2 Department of Physical Therapy Faculty of Medicine, University of British Columbia, 212 - 2177 Wesbrook Mall, Vancouver, BC V6T 1Z3 Canada; 3 Rehabilitation Research Program, Vancouver Coastal Health Research Institute, 4255 Laurel Street, Vancouver, BC V5Z 2G9 Canada

**Keywords:** Stroke, Telerehabilitation, Physical therapy, Fugl-Meyer, Lower extremity

## Abstract

**Background:**

The majority of stroke survivors experience motor impairment which benefits from rehabilitation treatment. Telerehabilitation, remote delivery of rehabilitation services, is a possible solution providing access to rehabilitation for stroke survivors living in rural areas or in situations like the COVID-19 pandemic where face-to-face treatment may be risky. However, valid and reliable motor impairment measures have not yet been established over a telerehabilitation platform. The Fugl-Meyer (FM) lower extremity assessment is widely used clinically and in research. Thus, the aim was to develop a modified FM for telerehabilitation (FM-tele) and assess the feasibility and preliminary agreement of FM-tele scores with the FM.

**Methods:**

Three phases were employed: phase 1 development, phase 2 feasibility, and phase 3 preliminary agreement. Literature review and consultation with clinicians were employed to develop the FM-tele. Community-dwelling individuals with stroke and FM evaluators were consulted to provide feedback via questionnaires on the feasibility of the FM-tele. To assess the preliminary agreement of the FM-tele, individuals with stroke participated in two sessions, one in-person and one via telerehabilitation. The standard version of the FM was administered during the in-person session. The FM-tele was administered in both sessions.

**Results:**

From phase 1, clinician consultation identified the following key principles: safety of the client, clear lower extremity visualization, and minimization of position changes which guided FM-tele development (*n* = 7). Feasibility was established in phase 2 where participants with stroke indicated that they felt safe and experienced ease following the standardized instructions, despite some technological concerns (*n* = 5). FM evaluators agreed that participants were safe and indicated effective standardized instructions. Phase 3 (*n* = 5) indicated preliminary agreement of the FM-tele compared with the FM.

**Conclusions:**

Participants with stroke and clinical consultation indicated the FM-tele developed for telerehabilitation is feasible. A lower extremity motor assessment tool for telerehabilitation is urgently needed for stroke survivors living in rural areas or when face-to-face visits are impossible. This pilot study provides preliminary support for a future study.

**Supplementary Information:**

The online version contains supplementary material available at 10.1186/s40814-021-00862-8.

## Key messages regarding feasibility

- *What uncertainties existed regarding the feasibility?* Using the standard Fugl-Meyer (FM) in telerehabilitation is in question as the current scale requires a therapist to apply manual resistance, test reflexes, and for stroke survivors to attain supine, sitting, and standing positions; transitions between positions increase fall risk and may reduce visualization of the lower extremities in telerehabilitation.
- *What are the key feasibility findings?* A modified version of the lower extremity portion of the FM (FM-tele) was created with the specific needs of telerehabilitation in mind. Through clinician consultation, key principles of safety, clear visualization of the lower extremities, and minimizing position changes guided the development of the FM-tele. Questionnaires indicated participants felt safe and experienced ease with following the standardized instructions, despite some technological concerns. Assessors agreed that participants were safe and indicated the standardized instructions were effective in guiding the participant throughout the session, with some need to adjust equipment setup to better visualize the participant’s lower extremity for some items. Preliminary agreement was found. Key feasibility findings include that the FM-tele we developed produces similar scores to the FM, even though the modified version only uses a sitting position, eliminated the reflex items, and used a resistance band instead of therapist-applied resistance.
- *What are the implications of the feasibility findings for the design of the main study?* The results of this feasibility study improve the design for the future study with participants using (1) their own towel vs. resistance band for one of the FM-tele items and (2) their own device and video-based platform with which they are familiar and meets Health Insurance Portability and Accountability Act (HIPPA) requirements to reduce technological barriers.

## Background

Stroke is a major cause of disability [1]. Nearly 85% of stroke survivors cope with long-term motor impairments that hinder daily function and decrease quality of life [2, 3]. These motor impairments benefit from rehabilitation which can be provided through telerehabilitation, the remote delivery of rehabilitation services. Many stroke survivors live rurally with reduced access to health services such as physical therapists [4]. Telerehabilitation is a possible solution to provide access to rehabilitation to stroke survivors living in rural areas or in situations like the COVID-19 pandemic where face-to-face treatment may be risky. Thus far, telerehabilitation for upper limb motor function following stroke is effective in assessments [5, 6] and treatments [6–8]; however, stroke survivors’ top priority is to regain community levels of mobility, which requires improvement in lower extremity function [9]. No lower limb stroke motor assessments have been developed to be delivered over telerehabilitation.

The Fugl-Meyer (FM) assessment is widely used in clinical and research initiatives, and it was the single-recommended assessment for measuring motor impairment from an international consensus on stroke outcome measures [10]. It is valid and reliable for measuring motor impairment after stroke [11]. Currently, no studies use telerehabilitation for lower extremity motor assessments after stroke, which could be partially attributed to the potential fall risk associated with performing a lower extremity assessment over telerehabilitation. The current lower extremity portion of the FM requires the patient to attain multiple positions: supine, sitting, and standing which could be challenging to safely instruct and capture over a webcam. Additionally, the scale requires a therapist to apply manual resistance and to test reflexes. Thus, the aim of this pilot feasibility study was (1) to develop a modified version of the FM suitable for telerehabilitation (FM-tele), (2) examine its feasibility to assess lower extremity motor impairment of stroke survivors, and (3) determine preliminary agreement between the FM-tele and FM.

## Methods

To develop and then test a FM-tele scale, a three-phase process was used: (1) development, (2) feasibility, and (3) preliminary agreement between FM-tele and the FM. Informed and written consent was obtained through the University of British Columbia Clinical Research Ethics Board (H19-02780) by each participant according to the Declaration of Helsinki prior to beginning data collection.

### Phase 1: Development

Literature review and consultation with clinicians were employed to develop the FM-tele and questionnaires for phase 2. The 7 team members had expertise in physical therapy and stroke rehabilitation (years of experience: 1 physical therapist (PT) > 20 years, 1 PT > 10 years, 5 PT students <2 years). Consultation sessions with clinicians centered on generating key principles to guide development of the new scale for telerehabilitation, and evaluating each item of the FM for criteria that the FM-tele would need to include.

#### FM-tele refinement

Information from the literature supplemented the consultation sessions to further refine the FM-tele (Additional file 1). Feedback sessions with the clinicians were conducted to refine the scale and ensure similar content relevance to the FM, standardized instruction clarity, and scoring clarity.

### Phase 2: Feasibility

Questionnaires for both assessors and individuals with stroke were developed alongside the FM-tele during the consultation sessions. To develop the questionnaires, clinical consultation sessions were employed which involved the same 7 team members with expertise in physical therapy and stroke rehabilitation (years of experience: 1 PT > 20 years, 1 PT > 10 years, 5 PT students <2 years). Based on the literature review and Telerehabilitation Best Practice Guideline recommendations by Blacquiere et al. (2017) and clinical consultation sessions, the questionnaires were developed iteratively alongside the FM-tele to ensure the items assessed the feasibility of the (1) real-time two-way video-conferencing platform and (2) whether the video-conferencing tool was easy to use and simple to operate [12]. The iterative process was considered complete when all clinicians were in agreement. The aim of the questionnaires was to gather overarching information regarding feasibility. Open-ended and Likert scale questions enquired about the effectiveness of the standardized instructions, benefits, and drawbacks of the FM-tele. The Participant Questionnaire consisted of 6 open-ended questions and 5 Likert scale questions, anchored as follows: 1 (completely disagree) to 5 (completely agree) (Additional file 2). The Assessor Questionnaire had 6 open-ended questions and 6 Likert scale questions, anchored as above (Additional file 3).

### Phase 3: Proportional agreement between items on the FM and FM-tele

#### Pilot study design

This study employed a test-retest design conducted at the GF Strong Rehabilitation Centre (Vancouver, Canada) and in participants’ homes via telerehabilitation. The first session involved the administration of the FM-tele and FM in person. Completing the two measures within the same session ensured data collection took place in the same environment and time of day to control for potential confounding factors. The second session, approximately 1 week later, administered the FM-tele in the participants’ home with telerehabilitation using eHAB (version 2.0.19, Neorehab, Queensland, Australia), a software designed for telerehabilitation. The one week delay was deemed sufficient time between sessions to minimize any real changes for the participant and adequate time so the assessors did not remember scores. Both sessions were conducted by the same assessor. All assessors were trained with training videos and in person training with an experienced research PT who is also a clinical instructor in a credentialing PT program.

#### Inclusion and exclusion criteria

To be eligible, participants were over 19 years old, > 6 months post-stroke, fluent in English, and able to communicate verbally. Participants were able to independently sit for 1 h. Participants with previous experience with telerehabilitation were excluded from this study.

#### Equipment

Equipment included an iPad (6th generation, software version 13.2, Apple Canada Inc., Toronto, Canada), iPad stand (model number STAN55232, Stouch), iPad charger, clip-on fisheye lens (model number B013W5QCI8, CamKix), red Theraband, and a setup handout. The clip-on fisheye lens served to widen the visual field captured by the camera lens, to fully visualize the participant. The setup handout provided a step-by-step outline of the home setup and sign in process for the online software.

#### In-person session

During the in-person session, both the FM and FM-tele were conducted by the same assessor. Participants were given a box with equipment and instructions required for the home session.

#### Telerehabilitation session

Telerehabilitation sessions were conducted online at participants’ respective homes and lasted approximately 1 h. Assessors communicated with participants online via eHAB [13, 14]. Participants followed the login instructions in a handout. If complications arose with setting up the iPad or accessing eHAB, participants were instructed to call the researchers at GF Strong Rehabilitation Centre for assistance. During the telerehabilitation session, only the FM-tele was conducted.

#### Analysis

The number of participant scores with agreement was summed for each item of the FM and FM-tele and reported in Table 1.

**Table 1:**
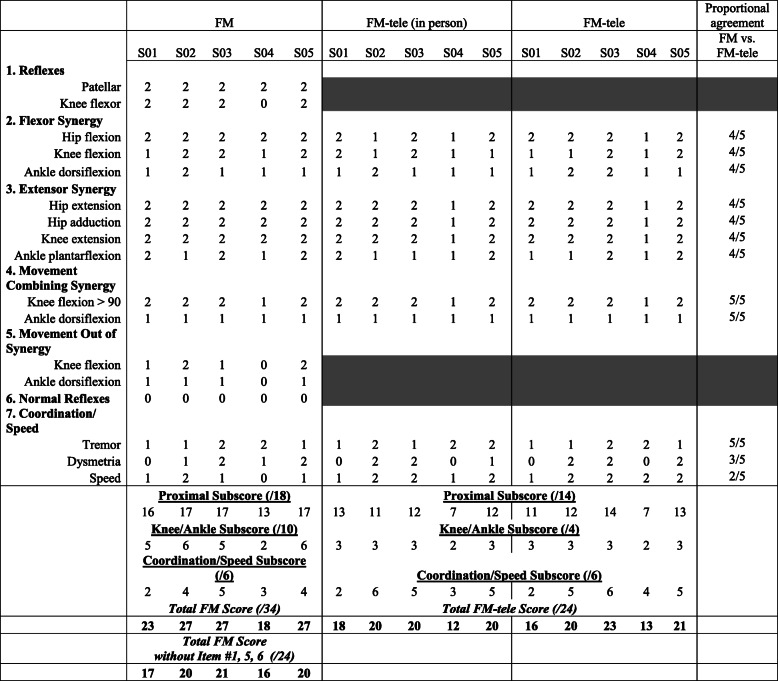
Individual participant scores on FM items

Dark shading indicates item not scored

*FM* Fugl-Meyer, *FM-tele* Fugl-Meyer telerehabilitation, *S* subject number

## Results

### Phase 1: Development

After a non-exhaustive literature search, no validated lower extremity measure of motor impairment specific for telerehabilitation was found. According to Kwakkel et al. (2017), an international consensus on stroke outcome measures recommends the FM for wide use in clinical and research initiatives to measure motor impairment after stroke [10], with work by Barbosa et al. [11] and Duncan et al. [15] showing high validity and reliability of the FM. The American Physical Therapy Association Neurology Section Task Force recommends use of the FM from acute to chronic phases of recovery after stroke [16]. The Canadian Stroke Best Practice Recommendations on Telerehabilitation were consulted to ensure consistency of the FM-tele with Best Practice Guidelines [12]. Specifically, telerehabilitation is recommended to be part of integrated stroke services for rehabilitation including (1) real-time two-way video-conferencing for patient assessment and (2) efforts to ensure the video-conferencing used is easy to use and simple to operate [12]. Cluster analysis by Woytowicz et al. (2017) and Rasch analysis by Woodbury et al. (2007) indicate that reflex testing did not contribute to scores on the FM upper extremity [17, 18].

Based on the literature review and after consultation sessions, the clinical team generated these key principles to guide the development of the FM-tele: (1) safety of the participant in their home, (2) need to visualize the entirety of the bilateral lower extremities to ensure scoring clarity, and (3) minimize changes in position to reduce participant fatigue and reduce the need to adjust the videocam. Also, items that required a therapist, such as palpation of tendons or application of therapist-applied resistance, were modified to ensure content relevance from the FM was encompassed within the FM-tele. The following rationale was employed on an item by item basis (Table 1, Additional file 1):

#### Item 1 Reflexes

Reflexes cannot be completed without a trained health professional; thus, this item was eliminated. Literature review identified that for the upper extremity FM, eliminating the reflex item did not considerably alter scores for impairment level [17, 18].

#### Item 2 synergistic flexor synergy

This item was modified from a supine to sitting position to be consistent with key principle #1 (safety), #2 (visualize entire leg), and #3 (minimal position changes). As palpation of the distal tendons could not occur, to ensure that knee flexion was active, clear visual observation confirmed active knee flexion in sitting. Participants were instructed to flex the hip, knee, and ankle joint fully while in sitting.

#### Item 3 synergistic extensor synergy

**Fig. 1:**
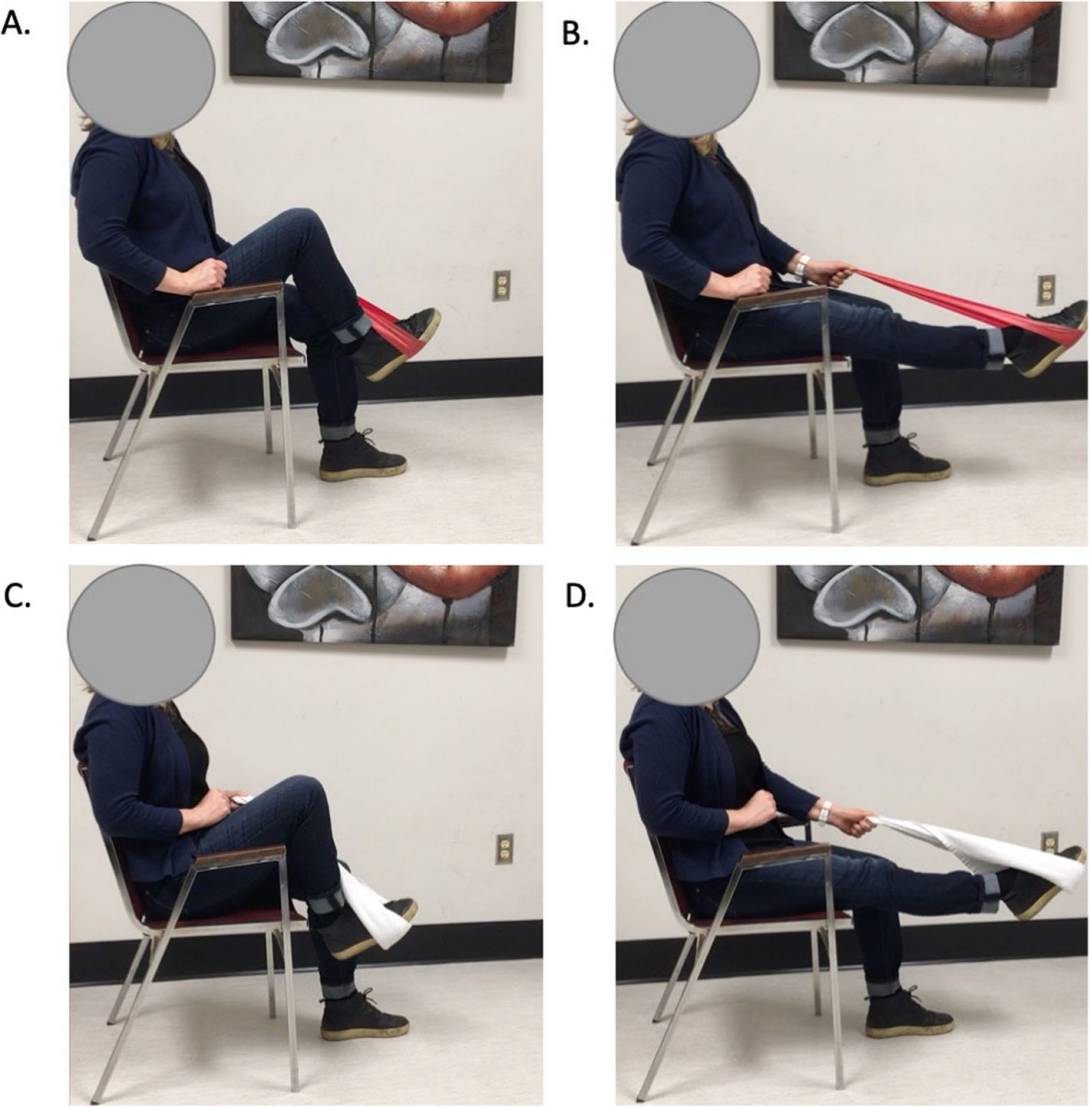
Start and end positions for Item 3 of the FM-tele. **A** The start position for the leg and theraband. The theraband is placed under the ball of the paretic foot with the non-paretic hand holding the ends of the theraband. **B** The end position for the leg and theraband. **C** The start position for the leg and towel for item 3 for the future study. The towel is placed under the ball of the paretic foot with the non-paretic hand holding the ends of the towel. **D** The end position for the leg and towel for the future study

This item was modified from a supine to sitting position to be consistent with key principle #1(safety), #2 (visualize entire leg), and #3 (minimal position changes). The FM requires resistance be applied to ensure movement is active and to evaluate both movement and strength. To mimic the effect of therapist-applied resistance, a resistance band was used for the FM-tele. The resistance band was approximately 1.25-m long without any knots or loops added to it. The ball of the foot was positioned in the middle of the length of the resistance band, and the participant held the ends of the resistance band with the non-paretic hand (Fig. 1A, B). From the position of full hip/knee flexion and ankle dorsiflexion in sitting, the participant was instructed to perform hip extension/adduction, knee extension, and ankle plantarflexion in a slow and controlled manner (i.e., over 3 s). If the participant demonstrated controlled and slow (≥3 s) movement, a resistance band was added under the ball of the foot, with the participant holding the end of the band in their hand. The participant then performs the same motion with this added resistance. To obtain a full score on a subitem (i.e., 2/2), the participant needed to demonstrate slow and controlled movement at that joint against the resistance band. If the participant could not assume the starting position, items #6 and #7 (combined knee extension/ankle plantarflexion) were assessed without hip extension/adduction (i.e., items #4 and #5 scored 0).

#### Item 4 movement combining synergy

As this item in the FM is already consistent with key principle #1 (safety), #2 (visualize entire leg), and #3 (minimal position changes) and is completed in sitting, there were no modifications made.

#### Item 5 movement out of synergy

In the FM, this item requires the participant to stand to assess knee flexion and ankle dorsiflexion while the hip is maintained at 0°. Based on literature review and clinical consultation, this item was deemed to be inconsistent with key principle #1 (safety) and #3 (minimal position changes). As a result, this item was eliminated as no modification in sitting could maintain the hip at 0° and still maintain content relevance with the FM.

#### Item 6 normal reflexes

This item was eliminated based on the same rationale as for Item 1.

#### Item 7 coordination/speed

This item was changed from supine to sitting position to be consistent with key principle #1 (safety), #2 (visualize entire leg), and #3 (minimal position changes). The same instructions as the FM were used.

Consequently, the FM-tele is scored out of 24 points (Additional file 1).

### Phase 2: Feasibility

Five community-dwelling individuals with stroke were recruited from the GF Strong Rehabilitation Research Program database (age = 63 ± 5.7 years; female *n* = 1; Table 2). These individuals had a range of impairment levels and were at least 6 months post-stroke. One caregiver who assisted a participant with activities of daily living also supported the participant with the questionnaire. Five assessors with clinical experience with stroke assessment (PT students <2 years’ experience), trained by the same research PT (>10 years of experience), completed the questionnaires regarding the FM-tele.

**Table 2:** Demographics

	Sex	Age (years)	Side of paresis	Months post stroke	Gait aid	Community ambulation
S01	M	72	Left	143	Cane	Yes
S02	M	63	Left	25	Single-walking pole	Yes
S03	M	58	Left	11	Single-walking pole	Yes
S04	F	58	Left	25	Powerchair, cane	No
S05	M	64	Left	45	None	Yes

Legend: *M* male, *F* female

#### Participant Questionnaire (Additional file 2)

No concerns were reported by any participants related to safety or following the standardized instructions given by the assessors. Technical concerns navigating the software were expressed by 100% of participants, with 60% of concerns being resolved during the session. The primary technical difficulties were related to the visual connection, such as screen freezing (reported by 40% of participants), and the inability to access clear audio on the platform (40%). Other logistical set up concerns were related to entering the personalized access codes to launch the online session (40%).

When asked to rank the ease of navigating the iPad system, participants gave an average score of 3.6 out of 5. Sixty percent of participants also reported that practice or familiarity with the iPad would have been beneficial, with one participant stating that they feel they would have benefited from more instructions on how to navigate the iPad. Furthermore, 80% of participants felt they would have had less difficulty navigating the device with repeated use. Sixty percent of participants reported that they either prefer or miss face-to-face interaction and 100% of participants either completely agreed or agreed that the overall physical therapy session was positive, despite the technical and logistical difficulties. Time efficiency of the session was the main reported benefit (40% of participants), with other perceived benefits including avoiding a commute (20%), familiarity with their environment (20%), and clarity of instructions (20%). When asked whether they would use telerehabilitation again in the future, all participants were either neutral, agreed, or completely agreed.

#### Assessor Questionnaire (Additional file 3)

Related to the FM-tele, all assessors agreed or completely agreed that the participants were safe during the telerehabilitation session; no adverse events such as falls or injuries occurred. Eighty percent of the assessors either agreed or completely agreed that the standardized instructions were effective in guiding the participant throughout the session. Deviations from the standardized instructions included adjusting the equipment setup to better visualize the participant. All of the assessors felt that they were able to give effective solutions when participants encountered problems with the assessment, which were mainly technological in nature (described below). Assessors also suggested that more detailed instructions for the environment setup and training for iPad use could have been beneficial for the participants.

All of the assessors found the software to be user friendly; however, all of them encountered malfunctions while using it. Most prominently, poor video quality due to video lag made the assessment difficult, especially when assessing coordination (Item 7). However, most assessors found that asking the participant to repeat the movement several times helped to perform an adequate assessment despite the poor video quality. Software glitches were also a common experience among the assessors, making establishing a timely and dependable connection with the participant challenging. This malfunction was often resolved by refreshing the eHAB platform or restarting the iPad. The audio failed to connect on two occasions, during which telephone was used to establish verbal communication with the participant instead. Common suggestions for improvement included ensuring a stable internet connection and developing software updates to minimize disruptions.

### Phase 3: Proportional agreement

The same five individuals with stroke from phase 2, participated in the two sessions for phase 3. Additionally, the same five assessors from phase 2, administered and rated the FM-tele for phase 3.

#### Range of scores

Scores on the FM ranged from 18 to 27 out of a possible 34. Scores of 0–19, 20–28, and ≥29 out of 34 are considered to reflect severe, moderate, and mild impairment, respectively [19]. Based on these cutoffs, one participant was severely impaired, with the other four participants moderately impaired. Scores on the FM-tele (in person) ranged from 12 to 20 out of a possible 24. Scores on the FM-tele ranged from 13 to 23 out of a possible 24. The data collected from all assessments (i.e., FM, FM-tele (in person), FM-tele) conducted in-person and over telerehabilitation are presented in Table 1.

#### FM and FM-tele

Table 1 presents the number of participant scores on each item in agreement across the FM and FM-tele. For the flexor synergy (Item #2), extensor synergy (Item #3), and movement combining synergy (Item 4), 4 out of 5 or 5 out of 5 participant scores were in agreement (Table 1). For Item #7 Coordination/Speed, dysmetria and speed were in agreement for 3/5 and 2/5 participants, respectively, possibly due to the poor video quality described in phase 2.

## Discussion

A modified version of the lower extremity portion of the FM was created with the specific needs of telerehabilitation in mind. After phase 1 through clinician consultation, key principles of safety (principle #1), clear visualization of the lower extremities (principle #2), and minimizing position changes (principle #3) guided the development of the FM-tele. Phase 2, feasibility of the FM-tele, indicated participants felt safe and experienced ease with following the standardized instructions, despite technological concerns. Assessors in phase 2 agreed that participants were safe and indicated the standardized instructions were effective in guiding the participant throughout the session, with some need to adjust equipment setup to better visualize the participant’s lower extremity for some items. Phase 3 indicated preliminary proportional agreement. These findings are promising, but more data are needed to confirm whether the FM-tele is a useful telerehabilitation motor assessment tool.

### Technological challenges

Technology posed the greatest challenge in this pilot study. Firstly, difficulties with Internet connections caused poor audio and video quality during most telerehabilitation sessions. This was a concern raised by both participants and assessors and indicates that having poor Internet connection can be disruptive for adequate motor assessment over telerehabilitation. Considering the broader implications, access to adequate internet connection may promote greater use of telerehabilitation services by clients living in rural communities. This also suggests that having a good Internet connection may act as a barrier to accessing rehabilitation services in remote populations. Offering a SIM card with preloaded internet connectivity is a potential way to address this barrier.

Secondly, difficulties with the eHAB software posed repeated complications when attempting to establish the participant-assessor online connection. Although troubleshooting eventually mitigated the difficulties, the assessment sessions often did not start on time. Improvements with participants’ familiarity with the equipment and software are another aspect that could improve the feasibility of using the FM-tele. As previously noted, participants voiced that more familiarity with the iPad would have been beneficial. Another alternative would be to use a device with which the participants are already familiar.

Thirdly, while the fish eye lens did increase the field of view, one participant lost the lens prior to the telerehabilitation session so data were collected without it. The assessor indicated visualization of the leg was not hampered as the participant sat farther back from the iPad. Encouragingly, the FM-tele appeared safe for participants. Despite participants setting up their own home environment and occasionally adjusting equipment throughout the session, no major safety concerns were raised by assessors or participants.

### Future study

Compared to the FM, in the FM-tele, 3 items were excluded (Items #1, 5, 6) and in 3 items, the body position is altered. This could be considered to be a large modification from the original FM, which may indicate the FM is not very feasible for telerehabilitation in its current form. Further, the results suggest that there is a need to develop outcome measures more suitable for assessment of the lower extremity for telerehabilitation. Thus, the FM-tele developed as part of this study requires establishment of its own psychometric properties prior to clinical use.

To improve development of a lower extremity motor assessment scale with items that perform well with telerehabilitation, the future study will explore the FM-tele by testing validity of inclusion and/or exclusion of the standing Item 5 (movement out of synergy, Table 1) in individuals with lower function. Also, since Item 7 (coordination) had the lowest agreement between FM and FM-tele (Table 1), the future study will ensure a more stable Internet connection for this item to test if agreement can be improved. The future study will also explore whether there is a ceiling effect due to the removal of the reflex items.

To this end, based on published reports [20] and the COSMIN recommendations and checklist [21], 50 or more individuals are needed to determine whether the FM-tele is similar to the FM and will use Kappa methodology to calculate reliability [21]. Thus, for the future study, a total of at least 60 individuals will be recruited to account for dropout.

To mitigate the technological challenges this pilot study uncovered, the future study will use a software platform the participant already is accustomed to using (ex. Zoom) that also meets the Health Insurance Portability and Accountability Act (HIPPA) requirements on the participant’s own device (ex. laptop, smartphone). Also, a fisheye lens will not be used to further reduce equipment barriers to assessment. We expect this will reduce technological challenges. To reduce equipment barriers to participation in the future study, participants will be asked to use a towel about 1.25 m long instead of resistance band for Item 3 (Synergistic Extensor Synergy). They will be asked to position the ball of their foot in the middle of the length of the towel and to hold the ends of the towel with the non-paretic hand (Fig. 1C, D). Requiring a resistance band to assess this item would necessitate purchase by the participant, or a clinician or researcher mailing or giving the item to the participant. Also, a towel providing resistance against the foot is sufficient for the assessor to observe controlled motion. To enhance future clinical utility, removing this barrier was deemed important. In the pilot study, a light resistance band was used to mimic the resistance due to gravity for this item; however, a towel is a common household item that can be used instead of a resistance band. Our participants were moderately to severely impaired, based on FM scores (Table 1) [19]. To increase generalizability, we will recruit from a range of impairment levels.

### Limitations

Although the findings show promise for the use of telerehabilitation in assessing lower extremity function in patients’ post-stroke, the following limitations were identified. One of the main limitations of this study is with generalizability: the results can only be used to inform and guide future studies. Our participants may be more experienced with technology than the general stroke population. This potential limitation is reduced with our planned future study by using technology participants are already familiar with. Also, our sample may not be representative of all stroke patients since our participants were living within an urban center as opposed to a rural setting because of the design of our study (1 in-person visit compared with 1 telerehabilitation visit). While these factors may limit generalizability, they do not impact the planned future study.

## Conclusions

The FM-tele we developed in this pilot study appears to be a promising assessment tool for telerehabilitation to underserved clients residing in rural communities; however, more research is required with a larger sample size. The FM-tele may be an efficient method of conducting lower extremity assessments for stroke survivors that may otherwise lack access to healthcare services in their communities. Development of rehabilitation assessment tools that can be used remotely is urgently needed for rural stroke patients with limited access to services.

## Supplementary Information

**Additional file 1.** Fugl-Meyer Lower Extremity Assessment for Telerehabilitation (FM-tele).

**Additional file 2.** Participant questionnaire.

**Additional file 3.** Assessor questionnaire.

## Data Availability

The data and materials collected in this study are all presented either in table or additional file.

## Abbreviations

FM: Fugl-Meyer
FM-tele: Fugl-Meyer telerehabilitation
