# Supplementary material for: Development and feasibility of a modified Fugl-Meyer lower extremity assessment for telerehabilitation: a pilot study
Source: Pilot Feasibility Stud. 2021 Jun 7;7:121. doi: 10.1186/s40814-021-00862-8 (PMC8182356; doi:10.1186/s40814-021-00862-8)
Supplement: Supplementary file 1 — Additional file 1. Fugl-Meyer Lower Extremity Assessment for Telerehabilitation (FM-tele). [file 40814_2021_862_MOESM1_ESM.docx]

Patient ID

Date

Assessed by

FUGL-MEYER LEG MOTOR ASSESSMENT FOR TELEREHABILITATION

| **Proximal** |
| --- |
| **I. Synergistic Flexor Synergy**  Instruct participant to flex his/her hip, knee and ankle joint fully while in sitting:   1. Hip flexion  - 0 (cannot be performed) - 1 (partial motion) - 2 (full motion)  1. Knee flexion  - 0 (cannot be performed) - 1 (partial motion) - 2 (full motion)  1. Ankle dorsiflexion  - 0 (cannot be performed) - 1 (partial motion) - 2 (full motion) |
| **III. Synergistic Extensor Synergy**  From the position of full hip/knee flexion and ankle dorsiflexion, instruct the participant to perform hip extension/adduction, knee extension and ankle plantarflexion in a slow & controlled manner (3 sec). If the participant demonstrates controlled and slow ($\geq$3 sec) movement, add resistance with a towel (length of 1 meter) under the ball of their test foot, holding the end of the towel in their hand. Instruct the participant to perform the same motion with this added resistance. If the participant cannot assume the starting position, assess items #6 and #7 (combined knee extension/ankle plantarflexion) without hip extension/adduction (i.e. items #4 and #5 scored 0).   1. Hip extension    - 0 (no motion or uncontrolled)    - 1 (little control and quick, $<$3 sec)    - 2 (controlled and slow, $\geq$3 sec, with towel) 2. Hip adduction  - 0 (no motion or uncontrolled) - 1 (little control and quick, $<$3 sec) - 2 (controlled and slow, $\geq$3 sec, with towel)  1. Knee extension  - 0 (no motion or uncontrolled) - 1 (quick and/or uncontrolled, $<$3 sec) - 2 (controlled and slow, $\geq$3 sec, with towel)  1. Ankle plantarflexion  - 0 (no motion or uncontrolled) - 1 (quick and/or uncontrolled, $<$3 sec) - 2 (controlled and slow, $\geq$3 sec, with towel) |
| **Knee/Ankle** |
| **IV. Movement Combining Synergy**  Sitting with knees free of chair/bedside, there should be slight knee extension   1. Knee flexion beyond 90^o^  - 0 (no active movement) - 1 (from slightly extended position, knee can be actively flexed but not beyond 90^o^) - 2 (knee flexion beyond 90^o^)  1. Ankle dorsiflexion  - 0 (no active dorsiflexion) - 1 (partial active dorsiflexion) - 2 (normal dorsiflexion) |
| **V. Coordination/Speed**  In sitting, have participant with eyes closed move their heel to the opposite knee 5 times in rapid succession.   1. Tremor  - 0 (marked tremor) - 1 (slight tremor) - 2 (no tremor)  1. Dysmetria  - 0 (pronounced or unsystematic dysmetria) - 1 (slight and systemic dysmetria) - 2 (no dysmetria)  1. Speed  - 0 (affected leg is 6 or more seconds slower than the unaffected leg) - 1 (affected leg is 2-5 seconds slower than the unaffected leg) - 2 (less than 2 seconds difference between the affected and unaffected legs) |
| **FUGL-MEYER SCORE**   1. PROXIMAL SUBSCORE (Q1-7) (0-14) 2. KNEE/ANKLE SUBSCORE (Q8-9) (0-4) 3. COORDINATION/SPEED SUBSCORE (Q10-12) (0-6) |
| 1. TOTAL FM TELEREHABILITATION SCORE (Q1-12) (0-24) |
