# Supplementary material for: Development and feasibility of a modified Fugl-Meyer lower extremity assessment for telerehabilitation: a pilot study
Source: Pilot Feasibility Stud. 2021 Jun 7;7:121. doi: 10.1186/s40814-021-00862-8 (PMC8182356; doi:10.1186/s40814-021-00862-8)
Supplement: Supplementary file 2 — Additional file 2. Participant questionnaire. [file 40814_2021_862_MOESM2_ESM.docx]

**Participant Questionnaire**

**Please answer the following questions below in regards to the telehealth session. For the questions involving a scale, please rate your level of agreement with each of the items. If you do not want to answer a question, you do not have to answer.**

1. The instructions you received on setting up the tablet in your home for the session were effective. Circle one.

| *1 2 3 4 5*  *Completely Disagree Neutral Agree Completely*  *Disagree Agree* |
| --- |

*Suggestions for improvement (optional)*

1. Did you encounter any problems with setting up the tablet for the session? *YES or NO* *.* If YES, what problems did you encounter?
2. Did you encounter any technical problems during the telehealth session? *YES or NO*. If YES, what problems did you encounter?
3. The tablet was easy to navigate. Circle one.

*
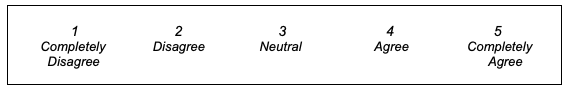
*

*Suggestions for improvement (optional)*

1. Did you experience any problems with the courier service used for receiving and sending the tablet? *YES or NO .* If YES, please explain
2. The instructions given to you by the physiotherapist were clear and easy to follow. Circle one.

*
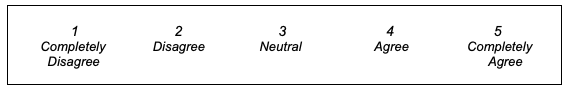
*

*Suggestions for improvement (optional)*

How safe did you feel during the session on a scale of 1-5?

*(1 = very safe; 5 = very unsafe)*

*Suggestions for improvement (optional)*

1. Your experience of the overall session was positive. Circle one.

*
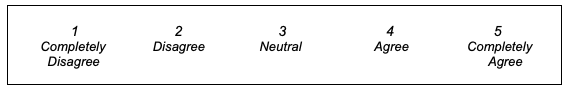
*

*Comments (optional)*

1. You would you use telehealth again to communicate with a physiotherapist or other healthcare professional if needed in the future. Circle one.

*
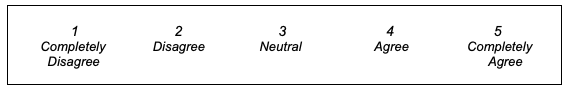
*

1. What were the most positive aspects of the session? *Open-ended*
2. What changes could be made to improve the experience and or delivery of the session? *Open-ended*
3. Other comments
