# Supplementary material for: Development and feasibility of a modified Fugl-Meyer lower extremity assessment for telerehabilitation: a pilot study
Source: Pilot Feasibility Stud. 2021 Jun 7;7:121. doi: 10.1186/s40814-021-00862-8 (PMC8182356; doi:10.1186/s40814-021-00862-8)
Supplement: Supplementary file 3 — Additional file 3. Assessor questionnaire. [file 40814_2021_862_MOESM3_ESM.docx]

**Assessor Questionnaire**

**Please answer the following questions below in regards to the telehealth session. For the questions involving a scale, please rate your level of agreement with each of the items.**

1. The video conferencing tool was user friendly and easy to use. Circle one.

*
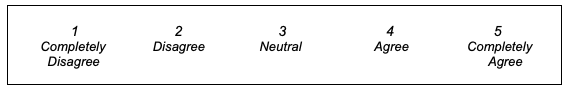
*

1. Did you encounter any problems with the video conferencing tool? *YES or NO*

If YES, please explain

1. You were able to adequately assess participants. Circle one.

*
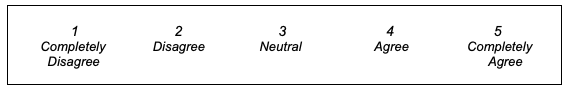
*

1. The standardized instructions were effective. Circle one.

*
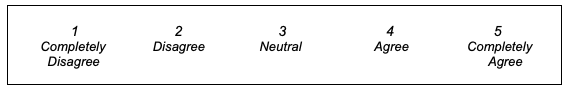
*

1. I did not have to change my instructions from the standardized version. Circle one.

*
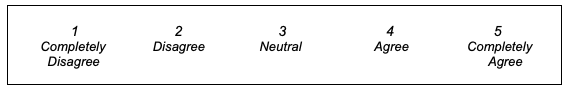
*

1. I felt the participants were safe during the telehealth session. Circle one.

*
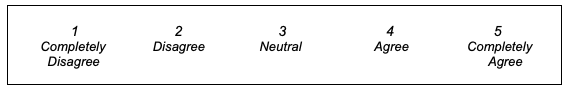
*

1. If participants encountered a problem with the assessment, I felt I was able to find a safe and/or effective solution. Circle one.

*
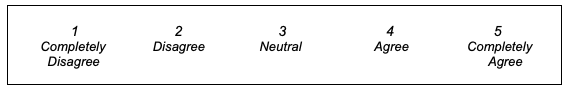
*

1. What components of the assessment were most challenging to conduct? *Open-ended*
2. What components of the assessment were easiest to conduct? *Open-ended*
3. What were the most positive aspects of the telehealth session? *Open-ended*
4. What changes could be made to improve the experience and or delivery of the telehealth session? *Open-ended*
5. Other comments
